# Sexual conflict explains the extraordinary diversity of mechanisms regulating mitochondrial inheritance

**DOI:** 10.1186/s12915-017-0437-8

**Published:** 2017-10-26

**Authors:** Arunas L. Radzvilavicius, Nick Lane, Andrew Pomiankowski

**Affiliations:** 1 Centre for Mathematics and Physics in the Life Sciences and Experimental Biology, University College London, Gower Street, London, WC1E 6BT UK; 2 Department of Genetics, Evolution and Environment, University College London, Gower Street, London, WC1E 6BT UK; 3 Department of Biology, University of Pennsylvania, Philadelphia, PA 19104 USA

**Keywords:** Heteroplasmy, Mitochondria, mtDNA, Paternal leakage, Sexual conflict, Uniparental inheritance

## Abstract

**Background:**

Mitochondria are predominantly inherited from the maternal gamete, even in unicellular organisms. Yet an extraordinary array of mechanisms enforce uniparental inheritance, which implies shifting selection pressures and multiple origins.

**Results:**

We consider how this high turnover in mechanisms controlling uniparental inheritance arises using a novel evolutionary model in which control of mitochondrial transmission occurs either during spermatogenesis (by paternal nuclear genes) or at/after fertilization (by maternal nuclear genes). The model treats paternal leakage as an evolvable trait. Our evolutionary analysis shows that maternal control consistently favours strict uniparental inheritance with complete exclusion of sperm mitochondria, whereas some degree of paternal leakage of mitochondria is an expected outcome under paternal control. This difference arises because mito-nuclear linkage builds up with maternal control, allowing the greater variance created by asymmetric inheritance to boost the efficiency of purifying selection and bring benefits in the long term. In contrast, under paternal control, mito-nuclear linkage tends to be much weaker, giving greater advantage to the mixing of cytotypes, which improves mean fitness in the short term, even though it imposes a fitness cost to both mating types in the long term.

**Conclusions:**

Sexual conflict is an inevitable outcome when there is competition between maternal and paternal control of mitochondrial inheritance. If evolution has led to complete uniparental inheritance through maternal control, it creates selective pressure on the paternal nucleus in favour of subversion through paternal leakage, and vice versa. This selective divergence provides a reason for the repeated evolution of novel mechanisms that regulate the transmission of paternal mitochondria, both in the fertilized egg and spermatogenesis. Our analysis suggests that the widespread occurrence of paternal leakage and prevalence of heteroplasmy are natural outcomes of this sexual conflict.

**Electronic supplementary material:**

The online version of this article (doi:10.1186/s12915-017-0437-8) contains supplementary material, which is available to authorized users.

## Background

Sexual reproduction in eukaryotes involves the fusion of haploid nuclei from both gametes. But the transmission of mitochondria and chloroplasts typically is limited to one of the gametes [1–3]. This pattern of uniparental inheritance (UPI) is nearly universal across eukaryotes, from isogamous protists with equal-sized gametes to animals and plants with extreme gamete-size asymmetry (i.e. oogamy and diminutive sperm). UPI is believed to facilitate purifying selection against deleterious mutations [4, 5], restrict intergenomic conflicts [6, 7] and limit heteroplasmy [8]. Cytoplasmic mixing, in contrast, reduces variation [5], impeding the efficacy of selection against defective organelles or selfish genetic elements [9, 10].

In isogamous organisms, nuclear genes restrict organelle transmission. Control can be “maternal” or “paternal”. Under “maternal” control, nuclear genes in one mating type destroy the mitochondria in the other mating type’s gamete at or after fertilization. In multicellular organisms, such maternal control is equivalent to the targeting and elimination of paternal mitochondria from sperm within the zygote after fertilization. In contrast, under “paternal” control, nuclear genes in one mating type destroy their own mitochondria during gametogenesis, which is equivalent to the exclusion or disabling of mitochondria during spermatogenesis (before entering the oocyte) in multicellular organisms. Under these definitions, the mating type that contributes the greater number of mitochondria to the next generation is maternal, and the mating type contributing less is paternal. Multiple attempts at modelling the evolution of asymmetric organelle inheritance have concluded that paternally controlled organelle destruction is precluded because of the lack of long-term linkage between the paternal nuclear genotype and its own mitochondria, as the cytoplasm is exclusively maternally inherited [3, 11–13]. For simplicity, these theoretical studies modelled the evolution of asymmetric inheritance in isogamous organisms [11–13]. But the conclusion holds true for multicellular organisms with anisogamy, as the same problem remains — a paternal nuclear gene that causes the exclusion of sperm mitochondria cannot build up linkage with maternally inherited organelles, as this relationship is re-set every generation.

Previous theoretical work suggests that maternally controlled elimination of paternal mitochondria should dominate in nature. Some empirical observations are consistent with this. In *Ascidian* tunicates, for instance, male organelles are prevented from entering the oocyte [14], and in the fungal plant pathogen *Ustilago maydis*, *lga*2 and *rga*2 genes expressed in mating type *a*2 are responsible for the selective elimination of the opposite mating type’s mitochondrial DNA (mtDNA) after fusion, at the same time protecting the mtDNA of the mating type *a*2 [15]. In other cases, however, paternal mtDNA is eliminated without any involvement of the maternal mating type. For instance, paternal control of cytoplasmic inheritance operates in *Drosophila melanogaster*, where mtDNA is actively degraded during spermatogenesis [16]. Similar elimination of mtDNA during spermatogenesis has been reported in fish and mice [17, 18]. Furthermore, in several cases the control of mitochondrial inheritance involves both parents. For instance, mitochondria in bovine and primate sperm are modified with ubiquitin during spermatogenesis, which serves as a signal for selective degradation after gamete fusion [19], and in the isogamous basidiomycete *Cryptococcus neoformans*, *SXI1α* in MAT*α* and *SXI2a* in MATa are both required for the UPI of MATa mitochondria [20]. In these cases, it appears that one mating type is responsible for tagging and the other for recognition and selective degradation of paternal organelles. A further example is maternally controlled autophagy that eliminates paternal mitochondria after fertilization in *Caenorhadbitis elegans* [21, 22], which has recently been demonstrated to be augmented by degradation of sperm mitochondria through paternal expression of the *cps-6* mitochondrial endonuclease during spermatogenesis [23].

These examples make it clear that paternal or dual control mechanisms are a common feature of spermatogenesis. They point to a glaring inconsistency in the current theoretical view that cannot explain paternal involvement in mtDNA transmission. The striking diversity of maternal and paternal mechanisms involved in restricting paternal transmission of mitochondria indicates repeated evolutionary turnover [1–3]. This presents an additional puzzle, given that current theory points to a universal advantage of organelle transmission from one sex. A further challenge to current theoretical views has come with the advent of next-generation sequencing, demonstrating that paternal leakage of mitochondria and persistent heteroplasmy are not as rare as traditionally thought [24–26]. Some degree of biparental inheritance (BPI) has been documented in diverse groups of animals, including mammals, arthropods, fish and birds, involving both interspecific [27, 28] and intraspecific matings [29–35]. These observations show that mechanisms preventing the inheritance of paternal mtDNA might be leaky at best or prone to evasion and failure. While biparental transmission in hybrid crosses could be explained by incompatibilities in molecular organelle tagging and recognition machineries, it is not clear whether paternal leakage within populations is a breach of a strict rule or is adaptive in its own right. The enigmatic case of doubly uniparental inheritance (DUI) in bivalve molluscs, where heteroplasmy in male somatic tissues is common, points towards the latter [36], but a theoretical explanation is lacking.

To understand the evolution of paternal control, turnover in mechanisms of control and the high prevalence of paternal transmission of mitochondria, we consider a model of a unicellular species with isogamy in which either mating type can control mitochondrial inheritance. We assume that purifying selection against mitochondrial mutations is the major force selecting for biased transmission of mitochondria [5]. In contrast to previous work, we assume that paternal leakage of mitochondria is a continuous evolvable trait and use an adaptive dynamics approach to specify the conditions under which strict UPI or varying degrees of paternal leakage are evolutionarily stable. We show that mitochondrial mixing can be selected under paternal control of cytoplasmic transmission and negative epistasis. The work provides the first theoretical explanation for the prevalence of paternal leakage, heteroplasmy and the repeated evolution of diverse mechanisms of UPI.

## Results

### Mathematical model

**Fig. 1:**
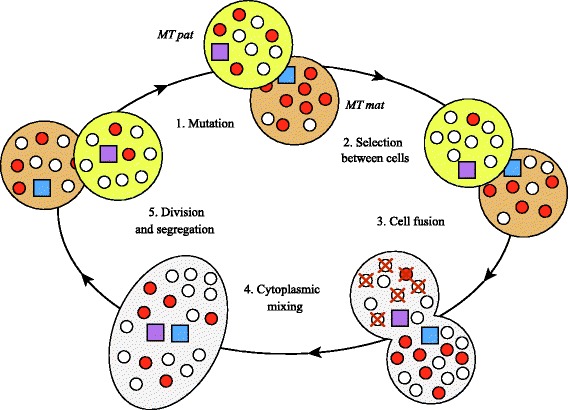
The model life cycle. The life cycle consists of discrete steps of (1) mitochondrial mutation (wild-type (*white*) to mutant (*red*) mitochondria, represented by *small circles*), (2) selection between cells, (3) random mating with cell fusion, (4) mitochondrial mixing and (5) division with random mitochondrial segregation. The paternal gamete (*yellow*) contributes only *πM* out of its *M* mitochondria to the zygote, shown here as elimination of some paternal mitochondria. This loss is made up by amplification of the maternal gamete (*brown*) contribution to (2 − *π*)*M* mitochondria. Note that a variety of sampling procedures for both maternal and paternal contributions did not affect outcomes (Additional file 3: Figure S3). The amount of paternal leakage *π* is controlled by either the maternal (*MTmat*, *blue square*) or paternal (*MTpat*, *purple square*) mating type nuclear genotype

We develop a mathematical model representing an infinite population of unicellular sexual haploid individuals, containing *M* mitochondria each (see Methods for a full description). Gametes are assumed to be isogamous, with differences in the number of mitochondria contributed to the zygote considered as an outcome of the model. The population life cycle consists of discrete generations with mitochondrial mutation, selection, mating, zygote division and random mitochondrial segregation (Fig. 1). Deleterious mutations occur within individual mitochondria at a rate *μ* per generation. Rather than studying mutations at individual loci, we treat *μ* as a genomic mutation rate to a deleterious state. Selection against defective mitochondria acts at the level of an adult cell with fitness expressed as $\omega =1-{\left(\frac{m}{M}\right)}^{\xi }$, where *m* is the number of mitochondrial mutations within a cell and *ξ* is the parameter determining the strength of epistatic interactions. For simplicity, this assumes weak selection on mutants when rare (i.e. *m* small), with strong deleterious effects as the number of mutant mitochondria increases (i.e. *m* → *M*). This conforms to empirical studies [37–39], which also suggest that the detrimental effect of new mitochondrial mutations increases steeply with heavier mutational load [37–39], so *ξ* ≥ 1 (i.e. negative epistasis). We assume that multiple mutations at different loci in the same mtDNA can be neglected (i.e. there are only two states, wild type and mutant), and that back mutations are so rare as to have negligible effect.

We assume that there are two mating types, arbitrarily labelled “maternal” *MTmat* and “paternal” *MTpat*. If there is maternal control, *MTmat* carries a nuclear allele controlling the number of mitochondria passed to the zygote from the paternal gamete. Under paternal control, *MTpat* carries a nuclear allele controlling the number of mitochondria transmitted to the zygote from the paternal gamete. We assume complete linkage of the UPI modifier to the mating type locus, as is the case in higher eukaryotes with two sexes, and is also known to be the case in some protists [15]. In either case, the zygote contains *πM* mitochondria from the paternal cell and (2 − *π*)*M* mitochondria from the maternal cell. The variable *π* measures the degree of paternal leakage of mitochondria. The model assumes that loss of paternal mitochondria is compensated for by greater maternal input to the zygote, and there is no direct fitness effect of *π*, only indirect effects that follow from the source of the mitochondria in the zygote (i.e. maternal or paternal). The diploid zygote then undergoes a two-step meiosis and segregates its mitochondrial population into daughter cells through random sampling. We consider small mutational changes in the value of *π* and use a numerical adaptive dynamics approach to find the continuously stable states with asymmetric transmission of mitochondria.

### With maternal control, strict UPI is the only asymmetric evolutionarily stable strategy (ESS)

**Fig. 2:**
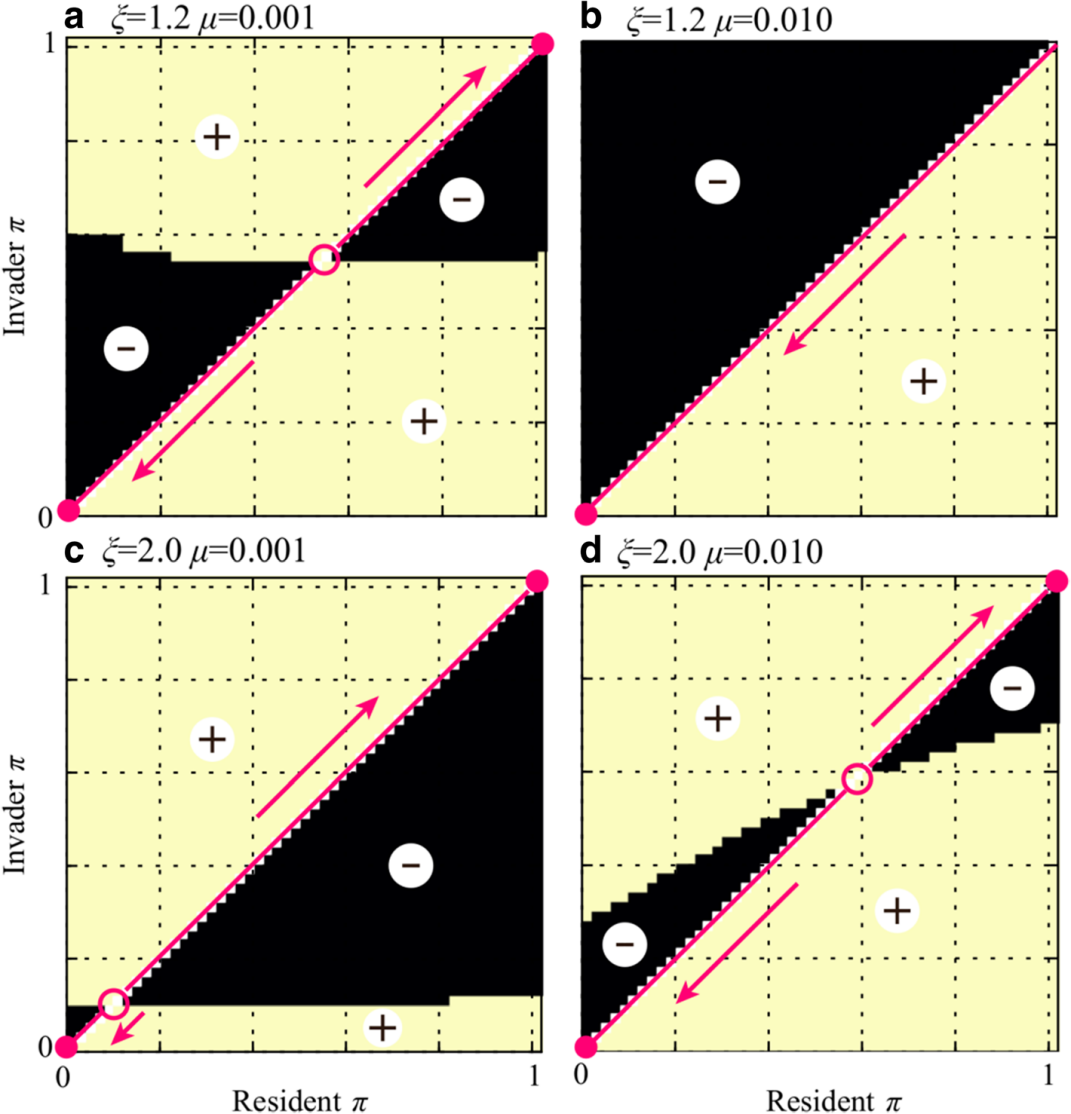
Pairwise invasibility plots for the maternally controlled asymmetric inheritance of mitochondria. Values of paternal leakage (*π*) for which invasion is successful are within the regions marked with “+”. *Arrows* show the direction of trait evolution assuming small mutational changes in *π*. **a** For weak epistatic interactions (lower *ξ*) and low mutation rates (*μ*), there are two evolutionarily stable states (*filled circles*), one at which mitochondria are symmetrically inherited from both gametes (*π* = 1), and the second at which there is full uniparental inheritance of mitochondria (*π* = 0). These are separated by a singular point between *π* = 0 and *π* = 1 which is an evolutionary repeller (*open circle*). **b** With higher mutation rates (*μ*), the zone of attraction to the asymmetric equilibrium (*π* = 0) increases, until the symmetric equilibrium is eliminated. **c**, **d** Increasing the degree of epistasis (higher *ξ*) increases the short-term benefit of mixing mitochondria and weakens the attraction of the asymmetric equilibrium (*π* = 0). The number of mitochondria was set to *M* = 50

In the conventional case, the maternal mating type *MTmat* regulates the contribution of paternal mitochondria to the zygote. Under maternal control, the invasion analysis recovers two boundary ESSs corresponding to complete uniparental (*π* = 0) and biparental (*π* = 1) inheritance, and an unstable singular point π* (0 < π* < 1) in between (an evolutionary repeller, Fig. 2a). Full UPI is the only ESS with asymmetric inheritance, while intermediate values of paternal leakage cannot be maintained. A population with BPI of mitochondria (*π* = 1) will not transition to UPI of mitochondria by exclusion of paternal mitochondria through small-effect mutations in *π* when mitochondrial mutation rates are low (Fig. 2). With high enough mutational rates and weak epistasis, BPI loses its local stability, and complete UPI (*π* = 0) becomes the sole evolutionary attractor (Fig. 2b). The range of values over which UPI is attractive is greater with increasing mutation rates (*μ*) and weaker epistatic interactions (*ξ* → 1) (Fig. 2a–d).

**Fig. 3:**
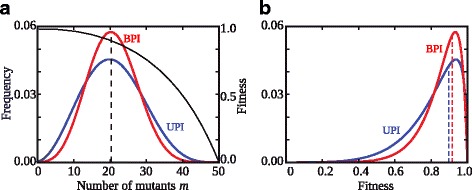
Changes in the variance of mutation number and fitness under maternal control. **a** Zygotic variance in mutation number is more constrained under biparental inheritance (BPI, *π* = 1) or higher values of *π* (*red*), as mixing mitochondria from two gametes increases the frequency of intermediate cytotypes compared to uniparental inheritance (UPI, *π* = 0) or lower values of *π* (*blue*). Note that the mode of inheritance does not alter the mean number of mutants (arbitrarily set at 20 mutants), only the variance in mutation frequency. The fitness function (*black curve*) is concave (epistasis *ξ* = 3), which assumes that a large number of mutants must accumulate before cell function is significantly undermined. Higher variance boosts the efficacy of purifying selection, allowing greater removal of mutant mitochondria in the long term. This favours lower values of *π*. **b** The distribution of zygote fitness given the frequency distributions in panel **a**. BPI has a short-term mean fitness advantage (*dotted lines*) because intermediate cytoplasmic states have higher fitness than the mean of the extreme states. Note that the variance in fitness is higher with UPI, as more individuals have higher as well as lower fitness

The local attraction towards either BPI or UPI under maternal control can be explained in terms of long- and short-term benefits. If the invader has a lower value of paternal leakage (*π*) than the resident, there is higher asymmetry of cytoplasmic inheritance and increased mitochondrial variance among the invaders (Fig. 3a). This boosts the efficacy of purifying selection and improves fitness over several generations, giving the lower *π* mutant a long-term advantage [4, 5]. Alternatively, the invader has a higher value of paternal leakage (*π*) than the resident, which increases the frequency of intermediate cytotypes. Under negative epistasis (*ξ* > 1) the fitness of the mix is higher than the mean of the two initial fitness values (Fig. 3b). More symmetric inheritance of mitochondria can therefore give the higher *π* mutant a short-term fitness advantage (Fig. 3b).

**Fig. 4:**
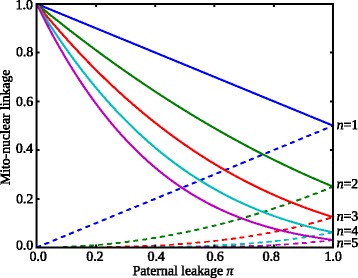
Strength of mito-nuclear statistical association (linkage). Linkage measured as the proportion of mitochondria after *n* generations that are identical by descent to those at generation *n* = 0. *Solid curves* represent linkage under maternal nuclear control of mitochondrial inheritance, while *dashed curves* correspond to paternal control. The two modes of control differ in the way that paternal leakage affects the strength of mito-nuclear associations over time. For maternal control, the linkage is ${\left(1-\frac{\pi }{2}\right)}^n$ and declines with *π,* as with increasing paternal leakage fewer maternal mitochondria are passed on to each offspring. For paternal control, the linkage strength is ${\left(\frac{\pi }{2}\right)}^n$ and increases with *π*, as with higher values of paternal leakage more paternal mitochondria are transmitted to the zygote

The evolutionary success of an invader is determined by the interplay between the long- and short-term effects and the degree to which the nuclear allele that controls *π* is associated with the resulting mitochondrial population. Long-term fitness effects are more relevant with strong mito-nuclear linkage, while short-term effects dominate under weak statistical associations between the two genomes. Since (by definition) the mother passes on more mitochondria, the maternal nuclear alleles are always strongly linked to the mitochondrial population, and this association strengthens as the paternal contribution of mitochondria falls (i.e. with lower values of *π*; Fig. 4). In addition, the established association strongly persists through further matings, especially as *π* declines, as there is less dilution by paternal mitochondria.

With maternal control, the singular point 0 < π* < 1 is located where the long- and short-term fitness effects are matched and the fitness landscape as experienced by a nearby mutation is virtually flat. An invader with a lower level of paternal leakage than the resident to the left of the singular point (i.e. *π* < π*) increases both the mitochondrial variance and the strength of mito-nuclear linkage (Fig. 4), and so invades due to the long-term fitness advantage and more efficient elimination of mitochondrial mutations. Conversely, to the right of the singular point (*π* > π*), an invader allowing more paternal leakage reduces both the variance and strength of the mito-nuclear association (Fig. 4) and thus benefits mostly from short-term effects. Successful invasion on either side of the singular point therefore makes it an evolutionary repeller under maternal control of cytoplasmic inheritance (Fig. 2).

### Paternal leakage is evolutionarily stable with paternal control of mitochondrial inheritance

In the reverse case of paternal control, mating type *MTpat* determines what fraction of its own mitochondria is discarded. Although the effect on the mitochondrial population of a zygote is the same as under maternal control, the evolutionary dynamics is radically different. The nuclear gene restricting paternal cytoplasmic inheritance is now patrilineally inherited and is therefore more weakly associated with its own mitochondrial population than the equivalent matrilineally inherited control allele (Fig. 4). The mito-nuclear association weakens with lower degrees of paternal inheritance (i.e. lower values of *π*) and dissipates more quickly across generations due to repeated dilution by maternal mitochondria. At the limit of *π* = 0, all mitochondria are inherited from the maternal gamete, and there is no association with the paternal nuclear allele (Fig. 4).

**Fig. 5:**
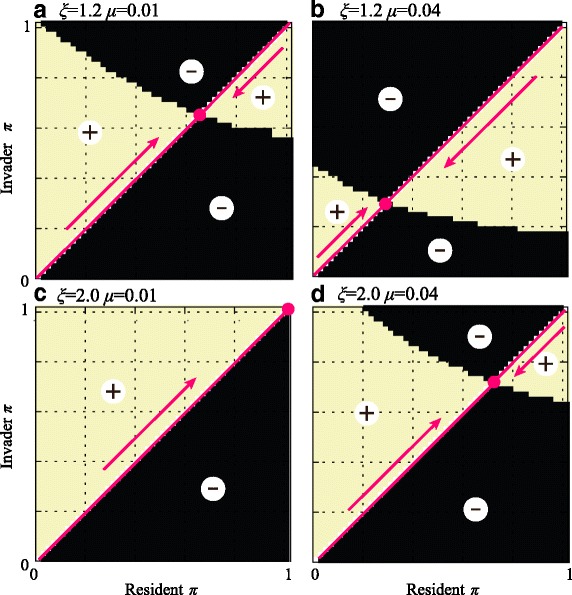
Pairwise invasibility plots for the paternally controlled mitochondrial inheritance. Values of paternal leakage *π* for which the invasion is successful are within the regions marked with “+”. *Arrows* show the direction of trait evolution, that is, the expected change in the value of *π* via recurrent invasion of mutants. **a** There is only one evolutionarily stable attractor at a singular point between *π* = 0 and *π* = 1. **b** Increasing the mutation rate favours an ESS with less paternal leakage (lower *π*), consistent with the need to purge deleterious mitochondrial mutations. **c**, **d** Increasing the degree of negative epistasis (higher *ξ*) increases the short-term benefit of mitochondrial mixing and favours higher levels of paternal leakage (higher *π*). The number of mitochondria was set to *M* = 50

With paternal control, our analysis again recovers a singular point corresponding to an intermediate level of paternal leakage (0 < π* < 1). However, this point is now an evolutionarily stable attractor (Fig. 5), contrary to the case of maternal regulation. This point is a continuously stable equilibrium [40], which is attractive across the full range of values of *π*. So, a population with complete BPI of mitochondria (*π* = 1) or one with UPI exclusively from the maternal gamete (*π* = 0) will evolve intermediate levels of paternal leakage. An ESS with more asymmetric inheritance (lower *π*) evolves with higher mutation rates (*μ*) and weaker epistatic interactions (*ξ* → 1) (Fig. 5). The evolutionary outcomes with maternal or paternal control only align when the mitochondrial mutation rate is sufficiently low that there is little long-term selection in favour of asymmetric inheritance, and when epistasis is sufficiently high that there is strong selection in favour of mixing, with BPI being the evolutionary outcome (Fig. 5d).

These results are explained once again by the balance between long- and short-term benefits of asymmetric inheritance, but this time with paternal control there is an opposite effect on the strength of mito-nuclear linkage. At the singular point π*, the long-term and short-term effects are in balance. An invader with *π* < π* increases the asymmetry in mitochondrial transmission and thereby increases variance. But in this case, it weakens the genetic association between the paternal nuclear allele and its own mitochondria, as there is greater dilution by the maternal mitochondria (Fig. 4). This is to the detriment of the invader. Conversely, although higher paternal leakage (*π* > π*) benefits from enhanced short-term effects, the reduction in variance undermines long-term outcomes, which is again harmful to the invader. This means that a singular point must necessarily be stable, since any deviation to either side is deleterious.

### Large mutational effects

The analysis above considers small mutational steps. Previous analyses considered large-effect mutations, but limited to two states, competing full UPI (i.e. *π* = 0) against full BPI (*π* = 1) [5, 8, 11, 41]. Here we allow arbitrary large-effect mutations and find different dynamics leading to polymorphisms where the mutant invades but does not completely replace the resident. Under maternal control these arise because the fitness advantage of an invading allele is subject to negative frequency dependence. As the invader becomes more common, selection in its favour declines (Additional file 1: Figure S1a). For example, an invader with a lower level of paternal leakage (*π*) than the resident benefits from long-term fitness effects. But due to random mating, these fitness benefits spread into the resident population. As the frequency of the invader rises, so does the fitness of the resident, reducing the invader’s advantage. Likewise, the fitness advantage of a mutant with higher *π* than the resident is greatest at low frequencies.

With small mutational changes in *π*, the negative frequency dependence is sufficiently weak that (under maternal control) the invader always goes to fixation and leads to unitary stable states at *π* = 0 or 1 (Fig. 2). But with large-effect mutations, a successful invader does not always replace the resident allele, spreading to an intermediate frequency instead. These polymorphic states always lie away from the main diagonal of the pairwise invasibility plots, and therefore cannot be reached via small mutational changes in *π* (Additional file 2: Figure S2). When a further invader is introduced into a polymorphic population, if it spreads, it drives out one of the existing alleles. The only stable endpoint is a population consisting of individuals with complete uniparental (*π* = 0) or biparental (*π* = 1) inheritance (Additional file 2: Figure S2). This means that evolution eventually leads to fixation, and a stable polymorphic ESS does not exist. This is true for both small and large mutational change.

In contrast, under paternal control the fitness advantage of a mutant increases with its frequency (Additional file 1: Figure S1b). Mutants causing increases or decreases in paternal inheritance of mitochondria either invade and go to fixation, or are lost. Polymorphisms do not result either for small or large mutational change.

More broadly, the assumption of large-effect mutations explains why earlier work precludes paternal control of mitochondrial inheritance and, by extension, the possibility of paternal leakage, or any competition between maternal and paternal control. These studies considered whether a discrete mutational state with complete restriction of paternal inheritance (*π* = 0) invaded the ancestral condition of BPI (*π* = 1) [5, 11, 12]. As there is no association of the paternal control nuclear modifier with its mitochondria under strict UPI (Fig. 4), it cannot be favoured by selection. In contrast, we show here that a continuous distribution of mutational states permits the association of paternal control with the inherited mitochondria over many generations, and this can favour asymmetric transmission with limited paternal leakage, depending on the mutation rate and epistasis.

## Discussion

Current theoretical views do not account for the active role of males in destroying their own organelles and cannot explain paternal leakage as anything more than a sporadic deviation from the rule of strict UPI, despite its common occurrence. In contrast to previous analyses [5, 11, 41], we allow paternal leakage to be a continuous evolvable trait. It is subject to indirect selection that acts against deleterious mutations in the mitochondrial population. Our analysis shows that paternally regulated asymmetric transmission of mitochondria evolves under a range of plausible parameter values (Fig. 5). We also find that paternal control is inherently associated with paternal leakage and persistent heteroplasmy.

A major conclusion of our work is that the evolutionarily optimal pattern of mitochondrial transmission critically depends on which mating type (or sex) controls the number of paternal mitochondria transmitted to the zygote. Maternal control favours strict UPI (*π*_mat_ = 0; Fig. 2), while paternal regulation favours an equilibrium with paternal leakage 0 < *π*_pat_ < 1 (Fig. 5). Strict UPI is favoured by the maternal mating type, due to its long-term effects increasing the efficacy of selection, and the strong association between mitochondria and nuclear genes for maternal control (Fig. 4). In contrast, males benefit from paternal leakage, since mitochondrial mixing increases the short-term mean fitness among their progeny, and linkage between mitochondria and nuclear genes for paternal control is weak and declines as the degree of asymmetry increases under paternal control, so long-term benefits are weaker (Fig. 4). The tension between short-term and long-term effects mirrors that seen in other sexual systems, for example, in the evolution of ploidy levels [42], gene duplications [43] and mate choice in yeast [44].

**Fig. 6:**
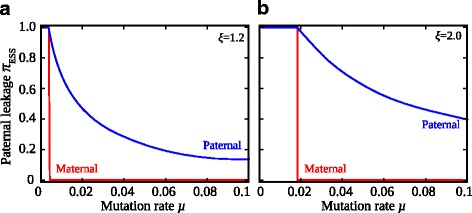
Sexual conflict over the inheritance of paternal mitochondria. The evolutionarily stable value of paternal leakage (*π*_ESS_) depends on whether the maternal (*red line*) or paternal (*blue line*) nucleus controls mitochondrial inheritance. This is shown for **a** weak epistasis (*ξ* = 1.2) and **b** strong epistasis (*ξ* = 2.0) across a range of mutation rates (*μ*). *π*_ESS_(mat) = 0 beyond a threshold mutation rate, favouring complete uniparental inheritance and no paternal leakage. In contrast, *π*_ESS_(pat) = 0 declines as *μ* increases and is always greater than zero, favouring some degree of mitochondrial mixing. The number of mitochondria per cell was set to *M* = 50

This dissonance means that the evolutionary outcomes under maternal and paternal control of mitochondrial inheritance rarely correspond (Fig. 6). As the mitochondrial mutation rate increases, these different equilibria become close but do not coincide, especially with stronger epistasis (Fig. 6a, b). This divergence implies there will be sexual conflict over the control of cytoplasmic inheritance leading to cycles of repeatedly evolving maternal and paternal control mechanisms. With reasonably high mutation rates, maternal control favours no transmission of paternal mitochondria (i.e. *π*_mat_ = 0) and establishment of strict UPI (*μ* > 0.005 in Fig. 6a or *μ* > 0.02 in Fig. 6b). This could be achieved by selective destruction of paternal mitochondria that are marked with ubiquitin, while protecting the maternal organelles, as is known to occur in several mammals [19]. Now consider a variant form of paternal control that affects the number of sperm mitochondria surviving within the zygote and subverting part of the maternal organelle-destruction machinery. For instance, this could be achieved by placing the ubiquitin tag on only a subpopulation of mitochondria during spermatogenesis or by favouring a variant of ubiquitin that is targeted less reliably for mitophagy in the zygote. According to our results, such a novel form of paternal control protecting some paternal mitochondria would spread, leading to the ESS with paternal leakage and persistent heteroplasmy (i.e. *π*_pat_ > 0). The short-term fitness advantage of mitochondrial mixing allows paternal control mutations to fix, even though paternal leakage reduces the variance in the mutation load, hinders purifying selection against defective mitochondria and poses a long-term fitness cost to both mating types. The paternally regulated state could persist, or the cycle could start again with a new maternal control mutation restoring strict maternal UPI. The sexual conflict over the control of cytoplasmic inheritance, with males benefiting from mitochondrial leakage and females favouring strict UPI, therefore predicts repeated turnover of the molecular mechanisms recognizing or protecting paternal organelles, accounting for the high diversity of maternal and paternal mechanisms controlling mitochondrial inheritance observed in nature [2, 3]. Note that the model used here assumes an infinite population, so there is no effect of drift. In finite populations, drift could lead to the spread of mutants affecting the degree of paternal leakage. The fate of such mutants would depend on the sex in which they were expressed, as described by our analysis.

The model we develop here explicitly deals with a unicellular organism. It does not consider a number of complexities inherent to multicellular organisms, like a germ line with distinct features in the two sexes and multiple rounds of mitotic division and segregation that precede the production of gametes. We have argued elsewhere that a number of the features of the germ line serve as additional mechanisms for the purging of deleterious mitochondrial mutations, producing a similar evolutionary advantage to UPI [45]. However, the general conclusions reached here regarding which parent controls mitochondrial inheritance, paternal leakage and turnover in mechanisms of cytoplasmic inheritance apply equally well to multicellular organisms, as the principles remain the same. Our modelling considers control of mitochondrial transmission from the paternal source. This makes sense when thinking about multicellular organisms, where sperm contain fewer mitochondria than eggs and are subject to maternal as well as paternal control mechanisms. In unicellular organisms, the designations “maternal” and “paternal” arbitrarily attach to the different mating types, and could easily be reversed. Again, both parents are involved in control of organelle inheritance [46]. Our modelling captures the two possibilities, namely that the mating types either control transmission of their own or of the other gamete’s mitochondria. There can be a range of complexities here as well, for example, where there are multiple mating types, long periods of asexual reproduction interspersed with occasional sex and diploidy of the adult forms [47–49]. In addition, selfing or inbreeding may alter selection in favour of UPI, as is known for selfish organelles [50, 51]. In our case, inbreeding raises linkage between nuclear and mitochondrial genes, particularly for paternally inherited nuclear genes, and this will break down the distinctiveness of selection on maternal and paternal control. All of these factors will have some impact on the selective pressures, but the principles we have uncovered remain the same.

Our results have implications for the evolution of oogamy as a means to control the extent of cytoplasmic mixing. Among its various benefits [45, 52], the development of a large female gamete packed with mitochondria can be viewed as one of the ways to enforce the highly asymmetric transmission of mtDNA. Assuming that the size of the sperm mitochondrial population cannot readily be increased, oogamy provides a reliable mechanism of asymmetric inheritance that is resistant to paternal counter-adaptations. Even if paternal mitochondria are not fully eliminated in the zygote, the potentially detrimental effects of heteroplasmy are restricted by the numerical preponderance of maternal mitochondria. We should not exclude the possibility, however, that paternal leakage could provide a more direct sex-specific benefit [53–55]. These sex-specific effects could be utilized through the preferential segregation of mitochondrial haplotypes into distinct somatic tissues, cases of which are known [56, 57]. The most striking example of such segregation occurs in bivalve molluscs with DUI, where M-type mitochondria are transmitted exclusively through the male germ line [36].

## Conclusions

The evolutionary stability of paternal leakage depends on how mitochondrial destruction is controlled. Under maternal control (destruction of mitochondria of the opposite mating type), strict UPI is favoured, while with paternal regulation (destruction of one's own mitochondria), paternal leakage is expected to evolve. Tension between selection on males and females leads to sexual conflict over the control of organelle inheritance, with different strategies favoured by the opposite sexes. This can explain the seemingly unstable evolutionary patterns of UPI with multiple origins and reversals, the numerous mechanisms involved in the asymmetric transmission of mitochondrial genes, the widespread occurrence of paternal leakage and prevalence of heteroplasmy in nature. Our analysis therefore offers a simple way of understanding the extraordinary variation in the patterns of mitochondrial transmission around the central tendency towards UPI.

## Methods

Consider an infinite population of haploid, unicellular organisms with two mating types, *MTmat* and *MTpat*, at equal frequencies. Note that this assumption means that we do not consider genetic drift at the population level. Each cell harbours *M* mitochondria during the haploid stage of the life cycle. A single nuclear locus controls the contribution of paternal mitochondria to the zygote, which can vary from strict UPI (no paternal leakage, *π* = 0) to complete biparental transmission (*π* = 1). The state of a population at any time *t* can be represented by the (*M* + 1) × 2 matrix **P** ^*l*(*t*)^, where index *l* denotes the mating type, *l* = 0 for *MTmat* and *l* = 1 for *MTpat*. The matrix element *P*_*m*,*j*_^*l*(*t*)^ then represents the frequency of cells of mating type *l*, with *m* mutant mitochondria and a nuclear state *j*. We consider allelic variation, *j* = 0 for the resident allele and *j* = 1 for a mutant invader, coding for different levels of paternal mitochondria transmission (*π*). These alleles are linked to the mating types, and they code for maternal control (linked to *MTmat*) or paternal control (linked to *MTpat*) of the contribution of paternal mitochondria to the zygote.

The life cycle consists of discrete and non-overlapping generations with distinct steps for mutation, selection and mating (Fig. 1). We consider two mitochondrial states, wild-type and a mutant state with impaired respiration. Wild-type mitochondria mutate at rate *μ*, but the reverse transition is ignored, as the probability of a back mutation is much lower. The state of the population at generation *t* and after the mutation step is therefore:

1 $$ {\mathbf{P}}^{l\left(t,1\right)}=\mathbf{U}{\mathbf{P}}^{l(t)};l=1,2, $$

where **U** is an (*M* + 1) × (*M* + 1) transition matrix. The matrix element *U*_*i*,*j*_ represents the probability that a cell with *j* mutant mitochondria will have *i* mutants after the transition,

2 $$ {U}_{i,j}=\left(\begin{array}{c}M-j\\ {}i-j\end{array}\right){\mu}^{i-j}{\left(1-\mu \right)}^{M-i};i,j\in \left[0,M\right]. $$

Mutation is followed by selection, with change in genotype frequencies according to their fitness values. We only consider selection between cells, not within the cell. In our model, cell fitness depends only on the mitochondrial part of the genotype; there are no mating-type specific fitness effects and no other differences in fitness between nuclear genes in different cells. The updated state of the population after selection is then:

3 $$ {\mathbf{P}}^{l\left(t,2\right)}=\frac{\left(\mathbf{Iw}\right){\mathbf{P}}^{l\left(t,1\right)}}{{\mathbf{w}}^{\mathrm{T}}{\mathbf{P}}^{l\left(t,1\right)}{\mathbf{u}}_2}, $$

where **I** is the identity matrix and **u**_2_ = (1, 1)^T^ is a column vector of ones. **w** is a column vector containing the *M* + 1 possible values of mitochondrial fitness,

4 $$ {w}_i=1-{\left(\frac{i}{M}\right)}^{\xi };i\in \left[0,M\right]. $$

*ξ* determines the magnitude of epistasis between mitochondrial mutations. *ξ* = 1 corresponds to the simplest case of additive fitness, where the fitness cost of each new mutation does not depend on the total mutational load. *ξ* > 1 means negative epistasis, where the fitness cost of several combined mutations is lower than expected under the additive model and increases with every new mutation. This corresponds to the mitochondrial threshold effects observed in experimental studies [38].

Surviving individuals of opposite mating types fuse at random, forming a population of diploid zygotes with 2 *M* mitochondria each. Let **z**_*gh*_ be a column vector with the *i*th element representing the frequency of zygotes containing *i* mutant mitochondria, and nuclear alleles *g* (inherited from the maternal gamete, *MTmat*) and *h* (inherited from the paternal gamete, *MTpat*). Zygote frequencies are then:

5 $$ {\displaystyle \begin{array}{l}{\mathbf{z}}_{00}=\left({\boldsymbol{\Phi}}^{\left(\pi \right)}{\mathbf{P}}_{\bullet, 0}^{0\left(t,2\right)}\right)\ast \left({\boldsymbol{\Psi}}^{\left(\pi \right)}{\mathbf{P}}_{\bullet, 0}^{1\left(t,2\right)}\right),\\ {}{\mathbf{z}}_{01}=\left({\boldsymbol{\Phi}}^{\left(\pi \right)}{\mathbf{P}}_{\bullet, 0}^{0\left(t,2\right)}\right)\ast \left({\boldsymbol{\Psi}}^{\left(\pi \right)}{\mathbf{P}}_{\bullet, 1}^{1\left(t,2\right)}\right),\\ {}{\mathbf{z}}_{10}=\left({\boldsymbol{\Phi}}^{\left(\pi \right)}{\mathbf{P}}_{\bullet, 1}^{0\left(t,2\right)}\right)\ast \left({\boldsymbol{\Psi}}^{\left(\pi \right)}{\mathbf{P}}_{\bullet, 0}^{1\left(t,2\right)}\right),\\ {}{\mathbf{z}}_{11}=\left({\boldsymbol{\Phi}}^{\left(\pi \right)}{\mathbf{P}}_{\bullet, 1}^{0\left(t,2\right)}\right)\ast \left({\boldsymbol{\Psi}}^{\left(\pi \right)}{\mathbf{P}}_{\bullet, 1}^{1\left(t,2\right)}\right),\end{array}} $$

where asterisks denote vector convolution (defined as [***a*** * ***b***]_*j*_ = ∑_*i* = 0_^*j*^ ***a***_*i*_ ***b***_*j* − *i*_) and the two transition matrices **Φ** ^(*π*)^ and **Ψ** ^(*π*)^ are included to implement bias in mitochondrial inheritance. We assume that the paternal mating type *MTpat* (**P** ^1^) contributes *πM* mitochondria through sampling without replacement, with (2 − *π*)*M* mitochondria coming from the maternal mating type *MTmat* (**P** ^0^) via random sampling with replacement ($\pi =0,\frac{1}{M},\dots, \frac{1-M}{M},1$). The two transition matrices then have elements:

6 $$ {\Phi}_{i,j}^{\left(\pi \right)}=\left(\begin{array}{c}\left[2-\pi \right]M\\ {}i\end{array}\right){\left(\frac{j}{M}\right)}^i{\left(1-\frac{j}{M}\right)}^{\left(2-\pi \right)M-i};i\in \left[0,\left(2-\pi \right)M\right],\kern0.75em j\in \left[0,M\right], $$

7 $$ {\Psi}_{i,j}^{\left(\pi \right)}=\left(\begin{array}{c}j\\ {}i\end{array}\right)\left(\begin{array}{c}M-j\\ {}\pi M-i\end{array}\right){\left(\begin{array}{c}M\\ {}\pi M\end{array}\right)}^{-1};i\in \left[0,\pi M\right],j\in \left[0,M\right]. $$

Alternative implementations of sampling had no effect on the conclusions of the study (Additional file 3: Figure S3). The cell cycle is completed by a two-step meiosis that restores the haploid adult state. For genotype frequencies at the start of the next generation we can write:

8 $$ {\displaystyle \begin{array}{l}{\mathbf{P}}_{\bullet, 0}^{0\left(t+1\right)}={\mathbf{F}}_2{\mathbf{F}}_1\left({\mathbf{z}}_{00}+{\mathbf{z}}_{01}\right),\\ {}{\mathbf{P}}_{\bullet, 1}^{0\left(t+1\right)}={\mathbf{F}}_2{\mathbf{F}}_1\left({\mathbf{z}}_{11}+{\mathbf{z}}_{10}\right),\\ {}{\mathbf{P}}_{\bullet, 0}^{1\left(t+1\right)}={\mathbf{F}}_2{\mathbf{F}}_1\left({\mathbf{z}}_{00}+{\mathbf{z}}_{10}\right),\\ {}{\mathbf{P}}_{\bullet, 1}^{1\left(t+1\right)}={\mathbf{F}}_2{\mathbf{F}}_1\left({\mathbf{z}}_{11}+{\mathbf{z}}_{01}\right),\end{array}} $$

where **F**_1_ and **F**_2_ are transition matrices for the first and second meiotic divisions implemented as mitochondrial sampling without replacement. Their corresponding elements are:

9 $$ {F}_{(1)i,j}=\left(\begin{array}{c}2j\\ {}i\end{array}\right)\left(\begin{array}{c}4M-2j\\ {}2M-i\end{array}\right){\left(\begin{array}{c}4M\\ {}2M\end{array}\right)}^{-1};i,j\in \left[0,2M\right], $$

10 $$ {F}_{(2)i,j}=\left(\begin{array}{c}j\\ {}i\end{array}\right)\left(\begin{array}{c}2M-j\\ {}M-i\end{array}\right){\left(\begin{array}{c}2M\\ {}M\end{array}\right)}^{-1};i\in \left[0,M\right],j\in \left[0,2M\right]. $$

To study the dynamics of the system, we consider the invasion of a mutant allele with a value of paternal leakage *π* different from the resident population. The new allele is inserted into the population at a small frequency *φ* = 0.005 at *t* = 500, so that ${\mathbf{P}}_{\bullet, 1}^{l(t)}=\varphi {\mathbf{P}}_{\bullet, 0}^{l\left(t-1\right)}$ and ${\mathbf{P}}_{\bullet, 0}^{l(t)}=\left(1-\varphi \right){\mathbf{P}}_{\bullet, 0}^{l\left(t-1\right)}$, and its evolution is tracked numerically until an equilibrium is reached. We consider all *M* + 1 possible values of the trait *π* and build the pairwise invasibility plots depicting the sign of the invasion fitness, i.e. the growth rate of the invader subpopulation when rare [58, 59]. These plots determine the expected evolutionary outcomes and stable strategies when the trait value changes in small discrete steps, but they also give insight into the dynamics of the system with large mutations [58, 59].

## Additional files

Additional file 1: Figure S1. The fitness advantage of the invader with *π* = 0.5 in a resident population with biparental inheritance (*π* = 1) is frequency dependent. (**a**) With maternal control, the fitness advantage of the invader declines with its frequency, leading to a stable equilibrium between 0 and 1. This can lead to a protected polymorphism where the mutant invades without completely replacing the resident, but these dimorphisms are not necessarily evolutionarily stable (Additional file 2: Figure S2). (**b**) Under paternal control of cytoplasmic transmission, the fitness advantage increases with allele frequency. The outcome of the invasion of paternal alleles is therefore always either fixation or extinction; i.e. dimorphic states cannot be established. In both cases the long-term advantage of asymmetric inheritance increases with the mitochondrial mutation rate *μ*, since asymmetric inheritance increases the efficacy of purifying selection against deleterious mitochondrial mutations. *M* = 50, *ξ* = 1.5. (DOCX 570 kb)

Additional file 2: Figure S2. Polymorphism under maternal control of mitochondrial inheritance, where the alleles code for two distinct values of paternal leakage *π*_1_ and *π*_2_. In the *yellow* regions, the invader allele coding for paternal leakage *π*_2_ invades and replaces the resident, and in the *black* regions, the invader allele coding for *π*_2_ cannot invade (see Fig. 2). In the coloured regions, the invader allele for paternal leakage *π*_2_ invades and reaches a protected polymorphism with *π*_1_. *Arrows* indicate the direction of evolutionary change in resident trait values, via recurrent invasions of a third mutant *π*_3_ when there is a protected polymorphism. Given that *π*_1_ < *π*_2_, invader *π*_3_ replaces resident *π*_1_ if *π*_3_ < *π*_1_; invader replaces *π*_2_ if *π*_3_ > *π*_2_, until the population reaches an evolutionarily stable polymorphism of coexisting *π*_1_ = 0 and *π*_2_ = 1 (*filled circles*). (**a**) The polymorphic states lie away from the main diagonal (except in the close vicinity of the repulsive singular point (*green circle*). (**b**) Increasing mutation rates favour an evolutionarily stable endpoint with higher frequency of *π*_1_ = 0, which under weak epistasis can completely displace the biparental allele. (**c**, **d**) Increasing the degree of negative epistasis (higher *ξ*) increases the short-term benefit of mixing mitochondria and reduces the frequency of the strictly uniparental *π*_1_ = 0 at the dimorphic ESS. (DOCX 1842 kb)

Additional file 3: Figure S3.Alternative sampling does not affect the qualitative outcome of the model. Pairwise invasibility plots (PIPs) for the maternal allele controlling paternal leakage *π*. (**a**) Sampling without replacement from both gametes. The maternal transition matrix is $$ {\Phi}_{i,j}^{\left(\pi \right)}=\left(\begin{array}{c}j\\ {}i-j\end{array}\right)\left(\begin{array}{c}M-j\\ {}\left[1-\pi \right]M-i+j\end{array}\right){\left(\begin{array}{c}M\\ {}\left[1-\pi \right]M\end{array}\right)}^{-1} $$ while the paternal matrix Φ_*i*,*j*_^(*π*)^ remains the same as in Eq. (7). (**b**) PIP produced with the original sampling method (Eqs. (6) and (7)). (**c**) PIP for sampling with replacement from both gametes. The paternal transition matrix is $$ {\Psi}_{i,j}^{\left(\pi \right)}=\left(\begin{array}{c}\pi M\\ {}i\end{array}\right){\left(\frac{j}{M}\right)}^i{\left(1-\frac{j}{M}\right)}^{\pi M-i} $$ while the maternal sampling remains the same as in Eq. (6). (**d**–**f**) PIPs for the paternal allele controlling paternal leakage *π*. (**d**) Sampling without replacement from both gametes. (**e**) PIP produced with the original sampling method (Eqs. (6) and (7)). (**f**) PIP for sampling with replacement from both gametes. (DOCX 1590 kb)
