# Supplementary material for: Sexual conflict explains the extraordinary diversity of mechanisms regulating mitochondrial inheritance
Source: BMC Biol. 2017 Oct 26;15:94. doi: 10.1186/s12915-017-0437-8 (PMC5658935; doi:10.1186/s12915-017-0437-8)
Supplement: Supplementary file 1 — The fitness advantage of the invader with π = 0.5 in a resident population with biparental inheritance (π = 1) is frequency dependent. (a) With maternal control, the fitness advantage of the invader declines with its frequency, leading to a stable equilibrium between 0 and 1. This can lead to a protected polymorphism where the mutant invades without completely replacing the resident, but these dimorphisms are not necessarily evolutionarily stable (Additional file 2: Figure S2). (b) Under paternal control of cytoplasmic transmission, the fitness advantage increases with allele frequency. The outcome of the invasion of paternal alleles is therefore always either fixation or extinction; i.e. dimorphic states cannot be established. In both cases the long-term advantage of asymmetric inheritance increases with the mitochondrial mutation rate μ, since asymmetric inheritance increases the efficacy of purifying selection against deleterious mitochondrial mutations. M = 50, ξ = 1.5. (DOCX 570 kb) [file 12915_2017_437_MOESM1_ESM.docx]

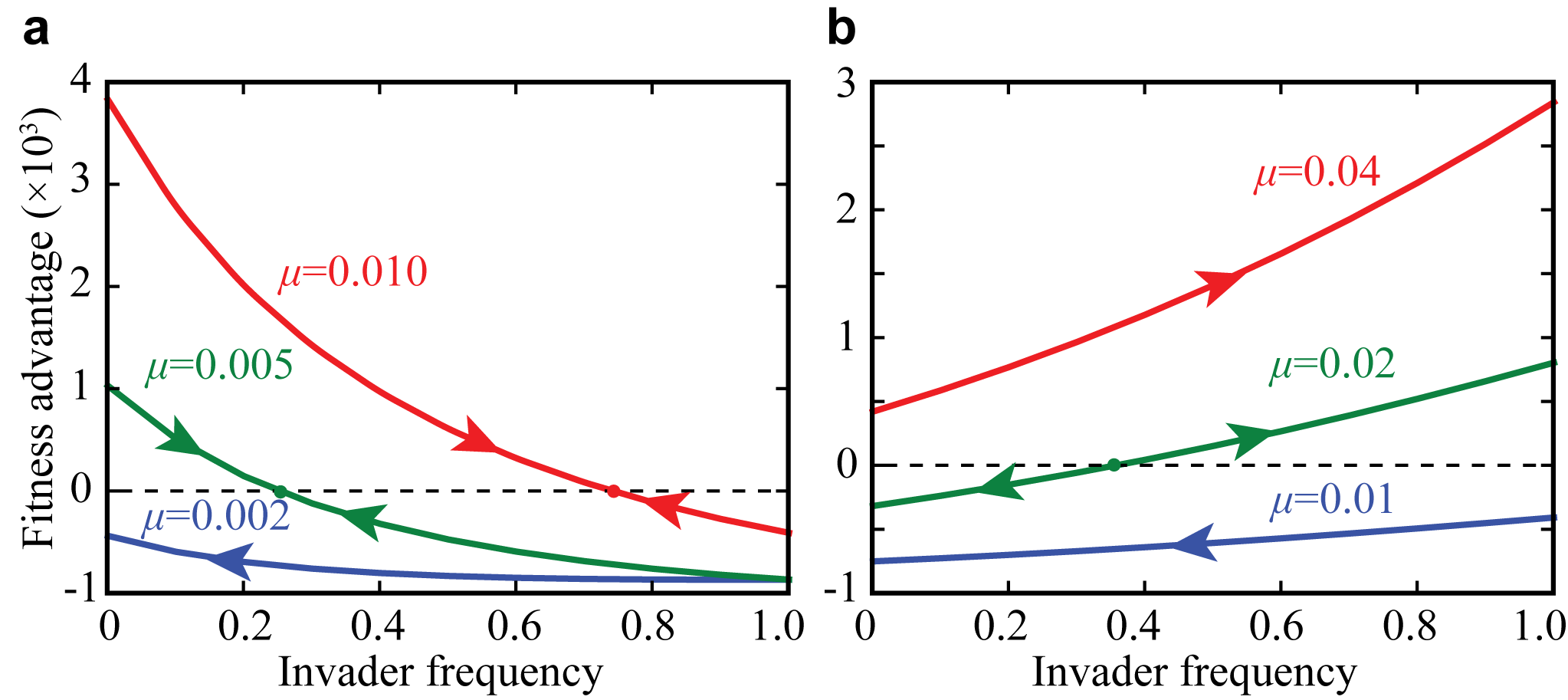


**Additional File 1: Figure S1**

The fitness advantage of the invader with $\pi=0.5$ in a resident population with biparental inheritance ($\pi=1$) is frequency-dependent. (**a**) With maternal control, the fitness advantage of the invader declines with its frequency, leading to a stable equilibrium between 0 and 1. This can lead to a protected polymorphism where the mutant invades without completely replacing the resident, but these dimorphisms are not necessarily evolutionarily stable (Supplementary Fig. 2). (**b**) Under paternal control of cytoplasmic transmission, the fitness advantage increases with allele frequency. The outcome of the invasion of paternal alleles is therefore always either fixation or extinction, i.e. dimorphic states cannot be established. In both cases the long-term advantage of asymmetric inheritance increases with the mitochondrial mutation rate $\mu$, since asymmetric inheritance increases the efficacy of purifying selection against deleterious mitochondrial mutations. $M=50$, $\xi=1.5$.

d 7). (**f**) PIP for sampling with replacement from both gametes.

**Sexual conflict explains paternal leakage, heteroplasmy and the extraordinary diversity of mechanisms regulating mitochondrial inheritance**

Arunas L. Radzvilavicius, Nick Lane, Andrew Pomiankowski
