# Supplementary material for: Sexual conflict explains the extraordinary diversity of mechanisms regulating mitochondrial inheritance
Source: BMC Biol. 2017 Oct 26;15:94. doi: 10.1186/s12915-017-0437-8 (PMC5658935; doi:10.1186/s12915-017-0437-8)
Supplement: Supplementary file 2 — Polymorphism under maternal control of mitochondrial inheritance, where the alleles code for two distinct values of paternal leakage π 1 and π 2. In the yellow regions, the invader allele coding for paternal leakage π 2 invades and replaces the resident, and in the black regions, the invader allele coding for π 2 cannot invade (see Fig. 2). In the coloured regions, the invader allele for paternal leakage π 2 invades and reaches a protected polymorphism with π 1. Arrows indicate the direction of evolutionary change in resident trait values, via recurrent invasions of a third mutant π 3 when there is a protected polymorphism. Given that π 1 < π 2, invader π 3 replaces resident π 1 if π 3 < π 1; invader replaces π 2 if π 3 > π 2, until the population reaches an evolutionarily stable polymorphism of coexisting π 1 = 0 and π 2 = 1 (filled circles). (a) The polymorphic states lie away from the main diagonal (except in the close vicinity of the repulsive singular point (green circle). (b) Increasing mutation rates favour an evolutionarily stable endpoint with higher frequency of π 1 = 0, which under weak epistasis can completely displace the biparental allele. (c, d) Increasing the degree of negative epistasis (higher ξ) increases the short-term benefit of mixing mitochondria and reduces the frequency of the strictly uniparental π 1 = 0 at the dimorphic ESS. (DOCX 1842 kb) [file 12915_2017_437_MOESM2_ESM.docx]

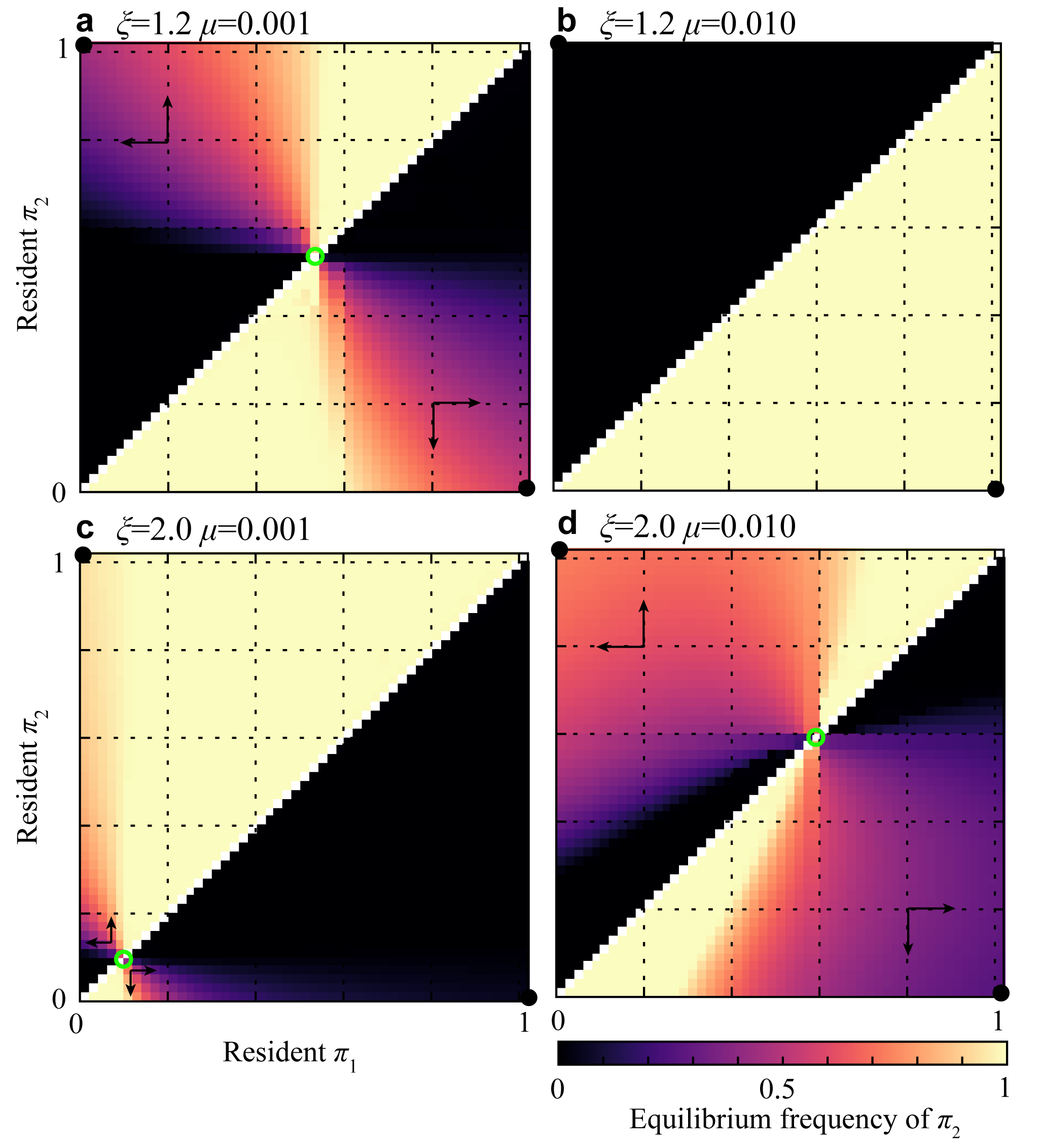


**Additional File 2: Figure S2**

Polymorphism under maternal control of mitochondrial inheritance, where the alleles code for two distinct values of paternal leakage $\pi_{1}$ and $\pi_{2}$. In the yellow regions, the invader allele coding for paternal leakage $\pi_{2}$ invades and replaces the resident; and in the black regions, the invader allele coding for $\pi_{2}$ cannot invade (see Fig. 2). In the coloured regions, the invader allele for paternal leakage $\pi_{2}$ invades and reaches a protected polymorphism with $\pi_{1}$. Arrows indicate the direction of evolutionary change in resident trait values, via recurrent invasions of a third mutant $\pi_{3}$ when there is a protected polymorphism. Given that $\pi_{1}<\pi_{2}$, invader $\pi_{3}$ replaces resident $\pi_{1}$ if $\pi_{3}<\pi_{1}$; invader replaces $\pi_{2}$ if $\pi_{3}>\pi_{2}$, until the population reaches an evolutionarily stable polymorphism of coexisting $\pi_{1}=0$ and $\pi_{2}=1$ (filled circles). (**a**) The polymorphic states lie away from the main diagonal (except in the close vicinity of the repulsive singular point (green circle). (**b**) Increasing mutation rates favour an evolutionarily stable endpoint with higher frequency of $\pi_{1}=0$, which under weak epistasis can completely displace the biparental allele. (**c**-**d**) Increasing the degree of negative epistasis (higher $\xi$) increases the short-term benefit of mixing mitochondria and reduces the frequency of the strictly uniparental $\pi_{1}=0$ at the dimorphic ESS.

**Sexual conflict explains paternal leakage, heteroplasmy and the extraordinary diversity of mechanisms regulating mitochondrial inheritance**

Arunas L. Radzvilavicius, Nick Lane, Andrew Pomiankowski
