# Supplementary material for: Sexual conflict explains the extraordinary diversity of mechanisms regulating mitochondrial inheritance
Source: BMC Biol. 2017 Oct 26;15:94. doi: 10.1186/s12915-017-0437-8 (PMC5658935; doi:10.1186/s12915-017-0437-8)
Supplement: Supplementary file 3 — Alternative sampling does not affect the qualitative outcome of the model. Pairwise invasibility plots (PIPs) for the maternal allele controlling paternal leakage π. (a) Sampling without replacement from both gametes. The maternal transition matrix is ${\Phi}_{i,j}^{\left(\pi \right)}=\left(\begin{array}{c}j\\ {}i-j\end{array}\right)\left(\begin{array}{c}M-j\\ {}\left[1-\pi \right]M-i+j\end{array}\right){\left(\begin{array}{c}M\\ {}\left[1-\pi \right]M\end{array}\right)}^{-1}$Φi,jπ=ji−jM−j1−πM−i+jM1−πM−1 while the paternal matrix Φi,j (π) remains the same as in Eq. (7). (b) PIP produced with the original sampling method (Eqs. (6) and (7)). (c) PIP for sampling with replacement from both gametes. The paternal transition matrix is ${\Psi}_{i,j}^{\left(\pi \right)}=\left(\begin{array}{c}\pi M\\ {}i\end{array}\right){\left(\frac{j}{M}\right)}^i{\left(1-\frac{j}{M}\right)}^{\pi M-i}$Ψi,jπ=πMijMi1−jMπM−i while the maternal sampling remains the same as in Eq. (6). (d–f) PIPs for the paternal allele controlling paternal leakage π. (d) Sampling without replacement from both gametes. (e) PIP produced with the original sampling method (Eqs. (6) and (7)). (f) PIP for sampling with replacement from both gametes. (DOCX 1590 kb) [file 12915_2017_437_MOESM3_ESM.docx]

**
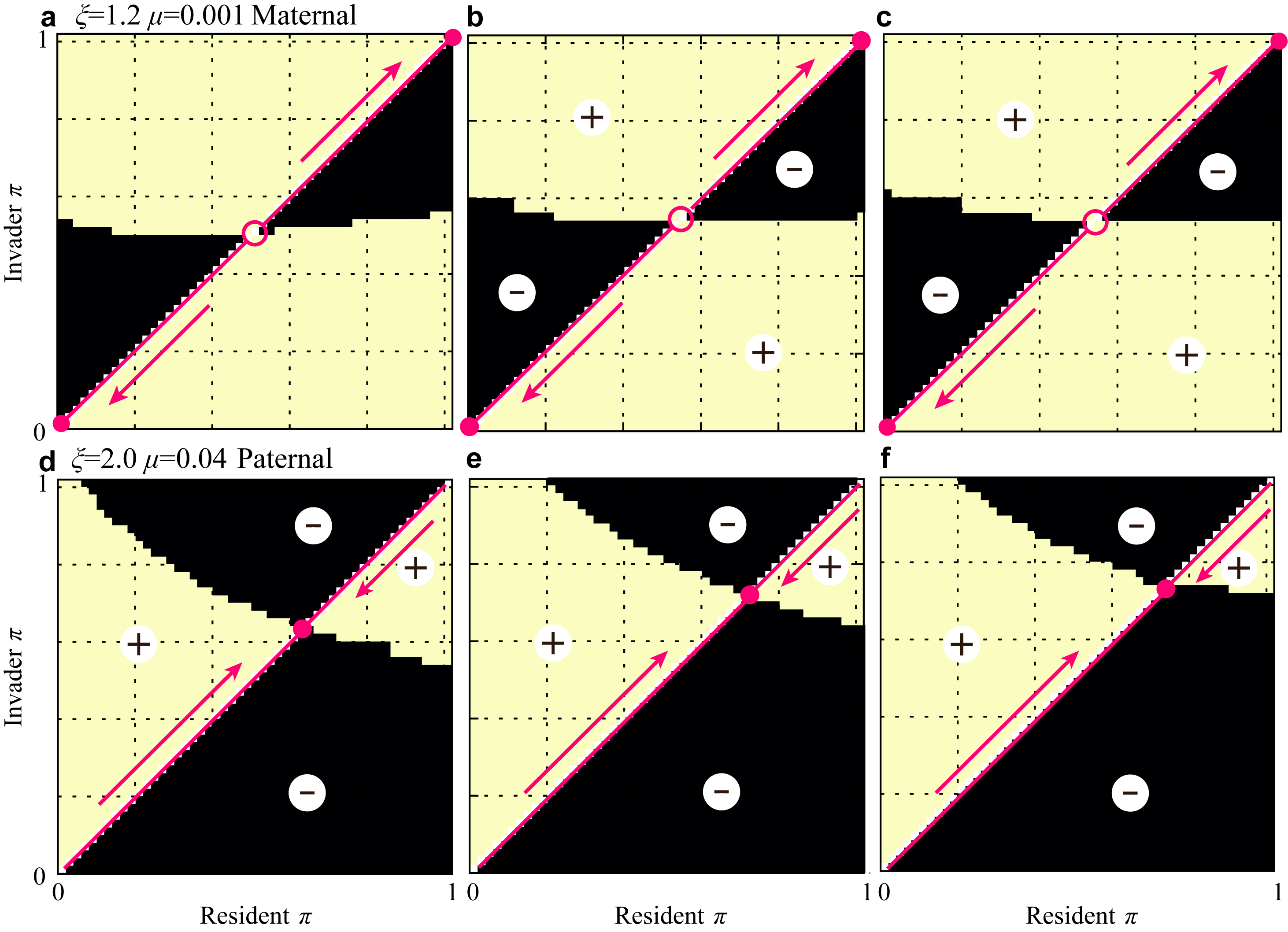
**

**Additional File 3: Figure S3**

Alternative sampling does not affect the qualitative outcome of the model. Pairwise invasibility plots (PIP) for the maternal allele controlling paternal leakage $\pi$. **(a)** Sampling without replacement from both gametes. The maternal transition matrix is $\Phi_{i,j}^{\left( \pi\right)}=\left( \begin{matrix} j \\ i-j \end{matrix} \right)\binom{M-j}{\left[ 1-\pi\right]M-i+j}{\binom{M}{[1-\pi]M}}^{-1}$while the paternal matrix $\Phi_{i,j}^{\left( \pi\right)}$remains the same as in eq. 7. (**b**) PIP produced with the original sampling method (eqs. 6 and 7). (**c**) PIP for sampling with replacement from both gametes. The paternal transition matrix is $\Phi_{i,j}^{\left( \pi\right)}=\left( \begin{matrix} \pi M \\ i \end{matrix} \right)\left( \frac{j}{M} \right)^{i}\left( 1-\frac{j}{M} \right)^{\pi M-i}$while the maternal sampling remains the same as in eq. 6. **(d-f)** Pairwise invasibility plots (PIP) for the paternal allele controlling paternal leakage $\pi$. **(d)** Sampling without replacement from both gametes. **(e)** PIP produced with the original sampling method (eqs. 6 and 7). (**f**) PIP for sampling with replacement from both gametes.

**Sexual conflict explains paternal leakage, heteroplasmy and the extraordinary diversity of mechanisms regulating mitochondrial inheritance**

Arunas L. Radzvilavicius, Nick Lane, Andrew Pomiankowski
